# What a difference a syllable makes—Rhythmic reading of poetry

**DOI:** 10.3389/fpsyg.2023.1043651

**Published:** 2023-02-13

**Authors:** Judith Beck, Lars Konieczny

**Affiliations:** Center for Cognitive Science, Institute of Psychology, University of Freiburg, Freiburg, Germany

**Keywords:** syllables, meter, reading, rhythm, poetry, top-down, bottom-up

## Abstract

In reading conventional poems aloud, the rhythmic experience is coupled with the projection of meter, enabling the prediction of subsequent input. However, it is unclear how top-down and bottom-up processes interact. If the rhythmicity in reading loud is governed by the top-down prediction of metric patterns of weak and strong stress, these should be projected also onto a randomly included, lexically meaningless syllable. If bottom-up information such as the phonetic quality of consecutive syllables plays a functional role in establishing a structured rhythm, the occurrence of the lexically meaningless syllable should affect reading and the number of these syllables in a metrical line should modulate this effect. To investigate this, we manipulated poems by replacing regular syllables at random positions with the syllable “tack”. Participants were instructed to read the poems aloud and their voice was recorded during the reading. At the syllable level, we calculated the syllable onset interval (SOI) as a measure of articulation duration, as well as the mean syllable intensity. Both measures were supposed to operationalize how strongly a syllable was stressed. Results show that the average articulation duration of metrically strong regular syllables was longer than for weak syllables. This effect disappeared for “tacks”. Syllable intensities, on the other hand, captured metrical stress of “tacks” as well, but only for musically active participants. Additionally, we calculated the normalized pairwise variability index (nPVI) for each line as an indicator for rhythmic contrast, i.e., the alternation between long and short, as well as louder and quieter syllables, to estimate the influence of “tacks” on reading rhythm. For SOI the nPVI revealed a clear negative effect: When “tacks” occurred, lines appeared to be read less altering, and this effect was proportional to the number of tacks per line. For intensity, however, the nPVI did not capture significant effects. Results suggests that top-down prediction does not always suffice to maintain a rhythmic gestalt across a series of syllables that carry little bottom-up prosodic information. Instead, the constant integration of sufficiently varying bottom-up information appears necessary to maintain a stable metrical pattern prediction.

## 1. Introduction

Regarding rhythm and timing, oral reading of unfamiliar conventional poetic language can be challenging at times as it includes, e.g., processing of an infrequent or irregular syntax, adjusting atypical phrase boundaries, or retrieval and prediction of stress and accent cues, etc (see Attridge, 1995; Hanauer, 2001; Carper and Attridge, 2003; Yaron, 2008). Thus, stress expectation management (Schmidt-Kassow, 2007; Schmidt-Kassow and Kotz, 2009a,b) may deviate gradually from lexical information, when the poem’s metrical grid and its rhythmic *gestalt* and melodic contour (see Schrott and Jacobs, 2011; Morgan et al., 2019; Scharinger et al., 2022) suggests so. Suprasegmental cues from preceding text material might even amplify this effect (compare Brown et al., 2015).

Metrically regular and rhymed language (MRRL) tends to be rhythmized differently compared to normal text/prose reading (Menninghaus and Blohm, 2020; de Arruda et al., 2022). For example, in both, silent and oral reading of MRRL, syllables play a central role as units of pronunciation (see Breen and Clifton, 2011, 2013; Breen, 2014, 2018; Beck and Konieczny, 2021). In oral reading, this is reflected in the systematic variation of syllable onset intervals (SOIs). Also, intensities for strong and weak syllables have been found to alter according to the respective meter (Fitzroy and Breen, 2020). Reading MRRL is thus characterized by pronounced articulatory gestures provoking a differential lengthening/shortening and intensifying of the smallest unit of speech, the syllable, hence causing an emphasized alternating stress distribution. The resulting phenomenon, that readers often quickly extract a beat from MRRL, and thereby project a meter onto upcoming lines, is an astonishing aspect of online processing during MRRL-reading. However, there has been little debate on the functional role of a specific composition of syllabic and phonemic material for establishing MRRL beat and meter.

To illustrate this phenomenon: After just a few words of reading this very sentence, you may have noticed that you have already started to establish a rhythmic pattern. You may have also automatically anticipated and predicted the upcoming stress distribution. And, right now, you are even reading the following sequence of *tack tack tack* as if they were real words. You probably have projected an alternating *strong-weak-(less) strong* stress onto them. Here, the *tack tack tack* sequence occurred as a one-time incident. But when reading longer texts containing more tacks and hence overall fewer prosodic cues, it can become difficult to update the stress expectation management during reading, even when the text imposes a concerted corset of meter and rhythm. Such is the case in conventionally metered and rhymed poetry:

As fragile as this breath could be
it flows tack tack – from A to C
and onwards, though tack tack tack see
this glimpse of whispers “*tack tack tack*”.

As fragile as this breath could be
it flows as air – from A to C
and onwards, though you cannot see
this glimpse of whispers “*to be free*”.

As can be seen from this example,^1^ other factors also contribute to the experience that poems bear musical quality (Blohm et al., 2021). Amongst them are melodic qualia (Menninghaus et al., 2018), the rhyme as a salient, verse-defining feature (e.g., Carminati et al., 2006; Wagner and McCurdy, 2010; Fechino et al., 2020) or alliterations (Lea et al., 2008). For all of these, as well as for meter, one structural principle of the MRRL is particularly noteworthy, namely *repetition* (compare Tierney et al., 2018a,b, 2021). It is only through the principle of structured repetition that all the rhythmic effects of the factors mentioned come to bear. In particular, this also applies to the perception and establishment of the metrical grid most frequently found in the poetic verses of a poem, resulting in a poem appearing to be iambic or trochaic in conception.

Based on findings by Woodrow (1909) on non-speech sounds and there tendency to be grouped either iambically or trochaically depending on duration or intensity, Hayes (1985, 1995) formulated the Iambic-Trochaic-Law (ITL) for speech-segments, according to which syllables that differ in intensity tend to be grouped as diads with initial prominence. In contrast, they are grouped into diads with final prominence if they differ in duration. However, there is more to it. When readers group a stream of stressed and unstressed syllables into metrical units that follow the principle of structured repetition, then whether it is perceived as trochaic or iambic depends on the factor that is held constant. For example, if only intensity is varied while syllable duration is constant, alternating segments are grouped trochaic (Hay and Diehl, 1999, 2007). What’s more, if perception is biased by an iambic or trochaic prime, the processing of successive segments can accordingly be affected (Fiveash et al., 2021), and this has supposedly biological underpinnings operating on abstract laws (compare Tabas et al., 2020). Additional factors that contribute to the realization of stress are pitch, spectral tilt, and in general, all shifts in vowel and/or consonant quality, resulting from adjusted articulatory gestures, e.g., vowel reduction or deletion, but also strengthening, gemination, or aspiration (compare Tilsen, 2019; Gordon and van der Hulst, 2020) – all mostly highly correlative of each other. Our study focuses on duration and intensity, which may well be considered the two most distinctive general features of emphasis. Other factors mentioned above are outside the scope of this paper.

For metrically regular, rhymed language (MRRL), a stronger and more systematic rhythmicity has been postulated (see Cummins and Port, 1998; Bröggelwirth, 2007). Testing the ITL for German language, Wagner (2010) examined the reading of poetry (trochaic, iambic, dactylic and “lied”) by comparing two groups, professional actors vs. non-professional jazz-choir singers. Results showed a clear durational contrast for iambs vs. trochees, with more pronounced lengthening of stressed syllables in iambs for both groups. However, subsequent analyses of phase relations suggests that independent of meter, a ratio of 3:2 (stressed:unstressed) was preferred. The author points out, that this pattern was more present in singers than in actors and concludes that the professional level of experience and the accompanying recitative expertise modulate the timing when rhythmizing MRRL. In addition, the data suggested that unstressed feet are more salient in iambic units than in trochaic ones. However, although for MRRL a more binary stance for stressing might hold true, a later comparison with prose (Wagner, 2012) revealed durational similarities, e.g., lengthening of the stressed syllable in iambs was found there, too. Still to date, the characteristics of the ITL are not yet sufficiently understood and most studies are based on decision-making or tapping experiments, mostly focusing on the perceptual mode (auditory channel), but rarely using oral production (for an overview see Wagner, 2022). For example, Wagner (2022) draws the attention to the specific functional relationship of prominence, i.e., salience, and duration, which he proposes do not necessarily “go hand in hand” (*ibid*., 269), and that both, speech production and speech perception need to be investigated. Although not MRRL was examined, but recorded (*bába bába bába*) and perceived (*ba ga ba ga ba ga*) sequences, his research findings suggest that listeners process words on the basis of two orthogonal dimensions, namely *duration* and *grouping*, of the respective auditory signal. Its interpretation is thus assumed to be understood as a relative relation of the two dimensions. The author concludes that “intensity and duration are generally poor cues for the distinction between iambs and trochees, but excellent cues for grouping and prominence” (*ibid*., 282). Wagner’s results highlight a key problem common for much of the literature related to ITL, namely the mixing of the physical properties of the sound signal itself with the cognitive phenomenon of projecting a derived abstract meter onto an incoming auditory signal and hence processing further input accordingly. For example, using an EEG oddball paradigm, Henrich and Scharinger (2022) had participants listening passively to pseudoword sequences while watching a silent movie. Pseudowords consisted of the disyllabic pattern *gugu* and were manipulated in pitch, however, not in duration or intensity, and also differed in foot (iambic/trochaic) or/and position of omission. Results showed that for iambs, omission of the second position led to stronger MMNs amplitudes compared to the first position omission, which was in line with their hypothesis. This pattern was correspondingly reversed for trochees. The omission on the strongly stressed first syllable of a trochee led to earlier MMNs than the weakly stressed first syllable of an iamb. For iambs, MMNs were even stronger when the strong syllable was omitted. This most likely can be explained by linking predictive processing of the (omitted) sound event (compare Tal et al., 2017) with the fact that for differentiation of events (i.e., by duration, intensity or pitch), an anticipated more salient event should be more crucial than the less salient one, presumabely always in relation to potentially preceding or subsequent silent pauses. Henrich and Scharinger (2022) interpret their results found for pitch in the predictive coding framework and state, that the MMNs elicited by omission mirror the “violation of a syllable-based prediction” and its prosodic quality (*ibid*, 8). They also report a significant MMN latency effect only for the first syllable, with shorter ones for trochees than iambs. They explain this effect with the ITL, which would expect higher pitch/intensities for foot beginnings and longer durations for foot endings (compare Crowhurst and Olivares, 2014; Crowhurst, 2020), as well as with the notion of possible articulatory habituation leading to a preferred trochaic patterning in German language (compare Wiese and Speyer, 2015; Wiese, 2016). However, in the described experiment by Henrich and Scharinger (2022), only pitch (see also Breen and Fitzroy, 2021) was under investigation but not intensity or duration.

Therefore, one of the leading questions of our study is, whether theoretical implications of the ITL, i.e., potential articulatory constraints such as intensity or duration, would nevertheless be applied to *tacks* during reading metrically regular, rhymed language (MRRL) aloud, which should actually all be stressed due to their onomatopoetic quality stemming from “ticktack” (tick-tock), which symbolizes the beat/ticking of a clock, as well as the “ck”-ending, which in German has a voiceless, short and hard *k*-sound. This also seems to add on the urge to stress the syllable by explicitly voicing the single vowel “a.” However, tack is no word by itself, yet it resembles characteristics of a word, but it does not carry individual semantic meaning. Importantly, in the rhythmic form of a poem *tacks* must be read partially unstressed according to the respective “governing” metrical grid of the poem conceptualized either iambically or trochaically. As a preliminary assumption regarding ITL, readers then should have already derived an abstract representation of the primary metrical unit - either iambic or trochaic - after reading just a few *tack*-less poem verses, and we should find *tacks* to differ accordingly in duration or in intensity. Hence, our focus was, firstly, on examining direct output of rhythmic MRRL-speech sounds, and secondly, online handling of MRRL-rhythm including violations of – as we would call it – *sound-scape*/*acoustical matrix* during oral reading. To our knowledge, no other study has tackled this specific problem yet. And, referring to the above described experimental results, another important question is to what extend results in relation to ITL might be confounded by effects of predictive processing shaped by an abstract metrical grid vs. the pure phonemic MRRL-material.

In other words, we were additionally interested in whether the *tack-*manipulations of the contrastive and consecutive order (Nolan and Jeon, 2014) of time-, respectively, pattern-bound MRRL-sounds could affect suspected top-down/bottom-up processes behind oral reading and might affect an assumed predictive processing of the main metrical figure, which has to be maintained and updated constantly throughout reading a poem in order to keep “the beat.” The principles of the predictive coding theory (PCT), (compare Rao and Ballard, 1999; Friston, 2008, 2010; Clark, 2013) have recently been applied to the processing and production of acoustic signals and transferred to the field of music (Koelsch et al., 2019; Vuust et al., 2022). For example, Vuust et al. (2022) had introduced the predictive coding of music model (PCM), whereby processing of music (perception, action, emotion, and learning) are postulated “recursive Bayesian processes, by which the brain attempts to minimize prediction error” (*ibid*., 289). Since processing music and language share circuits and overlap in brain mechanisms (see Fiveash et al., 2021), it is obvious to assume processes of predictive coding also for the processing of language, and especially of conventional poetic language. Thus, one can propose PCT/PCM as a theoretical framework in which possible interfering effects of *tacks* on establishing a certain meter – by perceiving a specific salience pattern in sounds, leading even to temporal beat distribution – can be discussed.

However, as debated in Engel et al. (2001) the classical dichotomy between top-down and bottom-up cannot be maintained (compare for music Pando-Naude et al., 2021; see also discussion Pourtois et al., 2008), which is relevant specifically to a cognitivist approach. Regarding the phenomenon of rhythm perception and beat induction (Honing, 2012, 2018) and applying it to metrically regular, rhymed language (MRRL), top-down hence may be captured best as a genre-driven stress expectation management on higher levels based on concrete sets of representative, albeit abstract units (Tabas et al., 2020), such as iambs or trochees. However, it is questionable, to which extend this is a conscious, internally generated, rather controlled act during online oral reading, potentially modulated by musical proficiency, and to which extend it is only existent and/or manipulable through interaction with the exogenous MRRL-input (i.e., bottom-up realization). Although the presented experiment only indirectly contributes to shed light on the assumed “interactive, bidirectional information exchange between levels of internal hierarchical systems” serving “to reconcile incoming information with internally generated predictions” (Rauss and Pourtois, 2013), it may well give insight on a syllabic-conceptual “threshold” in MRRL-reading, with respect to the actual oral output of the processing of multiple successive (presumably precision-weighted) “prediction errors,” elicited by a number of tacks, against a presumed strong “default” of prediction, i.e., either the iambic or the trochaic unit. This notion is captured by Vuust et al. (2022) formulation, that “prediction errors are useful only when things are predictable” (*ibid*., 289; compare also Ficco et al., 2021; Trapp et al., 2021).

In this context, an additional research question was whether musical proficiency can be a moderating factor or not for picking up a poem’s rhythmic figuration (compare Blohm et al., 2021, 2022) and “defend” the updating and maintaining of its main metrical grid against potential *tack*-interferences during oral MRRL-reading. Although we did not distinguish between basic auditory skills and musical expertise (for a discussion see Mankel and Bidelman, 2018; Tierney et al., 2021), the assumption holds that musically proficient readers have a prediction advantage because of precise timing due to musical practice and auditory training and thus also in anticipating speech-bound (MRRL) pauses (compare Patel, 2012, 2014; Turk and Shattuck-Hufnagel, 2014; MacIntyre and Scott, 2022). This should influence the application of durational and intensity patterns of syllables positively, which are the smallest unit of speech and most distinct rhythmic entity during oral reading of conventional poetry.

To sum up, we expect musically trained readers to extract a poem’s “beat” induced by the MRRL sound-gestalt easier than musically inactive readers, in accordance with the definitions given in Ravignani and Madison (2017) and Beck and Konieczny (2021). This in turn, should influence the temporal ratio, i.e., quasi-isochronicity (compare also Kotz et al., 2018; Ravignani et al., 2018; Aubanel and Schwartz, 2020), with which musical active readers encounter *tacks* during oral reading, differently so compared to inactive readers. Specifically, we use the SOI as well as mean intensity, to investigate rhythmic contrasts during MRRL reading, with a special focus on syllables which where substituted by the non-sensical syllable *tack*.

Additionally, we analyzed the rhythmicity of entire lines. The normalized pairwise variability index (nPVI) is a measure that provides an aggregated value of rhythmicity, and is often used in music analysis (Patel and Daniele, 2003; London and Jones, 2011). We used this measure to estimate the amount of rhythmic variation in each line. The nPVI can be computed from both SOIs and syllable intensities. Pairwise variability means that strong variations between adjacent syllables leads to high nPVI scores. Both the iambic and the trochaic should result in elevated nPVIs, because both imply an alternating pattern of stresses.

Our hypotheses for SOI and intensities were:

For regular syllables:

*H1*: Reading patterns resemble speaking patterns (Gagl et al., 2022), where stressed syllables are often longer and louder than unstressed syllables (for English see Carter and Clopper, 2002). We therefor expect SOIs to be longer, and intensities to be higher for strong syllables than for weak syllables (simple stress hypothesis).

*H2*: When poems are read aloud, “acoustic” sequences must be produced from the “silent” text material. If the acoustic signal is generated along the lines of how it is decoded, then properties established by basic iambic-trochaic-law effects in perception (Hayes, 1985, 1995) should also apply to oral production. We hence expect syllable strength to be expressed more by *intensity* differences in trochaic meter, and more by SOI differences in iambic meter (ITL hypothesis).

*H3*: Metric grids consist of syllables clustered into metrical units. In the iambic grid, these units are comprised of dyads of an unstressed syllable followed by a stressed syllable, and vice versa in trochaic grids, where a stressed syllable is followed by an unstressed one. When metrical units are produced as pronunciation units, we expect an increased likelihood of separating pauses, resulting in prolonged syllables onset intervals for the final syllable in the unit. The *metrical unit hypothesis* hence predicts an *interaction of meter and stress*: In iambic meter, the stressed syllable becomes lengthened even more, whereas in trochaic meter, it is the unstressed syllable that is lengthened (metrical unit hypothesis).

*H4a*: According to the OPERA hypothesis (Patel, 2011, 2012, 2014), years of training in musical skills should be associated with an improvement in temporal precision. We therefore expect musicality to be a modulating factor, particularly for SOIs, such that the effect of stress on SOIs should be more pronounced for musically active readers.

*H4b*: To compensate the lack of temporal precision, musically inactive readers might prefer intensity to express stress, such that the effect of stress on intensity should be more pronounced for musically inactive readers.

*H4c*: On the other hand, musically inactive readers might be less effective in expressing stress *in general*, such that the effect of stress on intensity should be less pronounced for musically inactive readers.

For tacks:

*H5*: When top-down processing prevails in the absence of distinctive bottom-up information on tacks, iambic and the trochaic speaking patterns should be projected onto tack syllables, such that SOIs and intensities for “tacks” should reflect the stress pattern of the metrical grid, as they do for regular syllables.

*H6*: If bottom-up processing prevails over top-down processing, “tacks” should be leveled out, i.e. they should be pronounced more similarly to each other, resulting in similar SOIs and intensities, regardless of stress.

*H7*: The syllable “tack” itself has a tendency to be pronounced as stressed, due to its phonetic properties. If top-town predictions are projected onto tacks, readers might therefor experience a conflict whenever the metrical grid suggests tacks to be unstressed. This interference can result in “paradoxical” SOI lengthening for weak tacks.

*H8*: Corresponding to H2, we expect that stress is expressed more via intensity differences in trochaic poems, and more by SOI differences in iambic poems. This should also apply to tack-syllables, such that the effect of stress on intensity should be greater in trochaic poems, whereas the effect of SOIs should be greater in iambic poems. This hypothesis amounts to a comparison between two two-way interactions (stress × meter, for intensity and SOI).

*H9*: Rhythmic reading requires the permanent alignment of bottom-up and top-down information to ensure the maintenance of the metric grid. Because tacks lack distinctive bottom-up information about stress, the top-down projection of a metrical grid could fade away with an increasing number of tacks in the same verse, and it should become increasingly difficult to maintain the metrical grid. That would be reflected in an interaction of *stress* and *tack index/tacks per line*.

*H10a,b,c*: Consistent with H4a for regular syllables, we expect that musically active readers will be better at maintaining the metrical grid, even in the absence of supporting bottom-up information. For tack-syllables, we also expect interactions between *musical* and *stress* corresponding to H4a, b, and c.

*H11*: We expect musically active participants also to be less affected particularly when they encounter multiple tacks within a line. This predicts an interaction of *musical*, *stress*, and *tack index*/*tacks per line*.

Hypotheses for rhythmicity, aggregated per line:

*H12*: If syllable-*stress* is reflected in SOIs and intensities varying between strong or weak syllables, we also expect to find line-based nPVI effects for SOIs and intensities for predictors that show a modulating effect on/interaction with stress, such as meter and musicality. If confirmed, the nPVI would thus provide a simpler aggregated measure – allowing for models with fewer predictors – that could still capture these effects.

## 2. Materials and methods

### 2.1. Participants

In total, 17 participants (11 women, 6 men; *M* age = 29.53; *SD* age = 13.37, range: 20–69 years) took part in the reading experiment, all were native speakers of German. In exchange, subjects received course credits or could sign up for a raffle. All participants gave written consent before the experiment started and the study was conducted in accordance with the “Ethical Principles for Medical Research Involving Human Subjects” (Declaration of Helsinki, 1964). Data of four participants had to be excluded because of technical mistakes during data collection. The remaining data of 13 participants (8 female, 5 male; *M* age = 31.85; *SD* age = 14.58, range: 21–69 years) were used for the analysis.

### 2.2. Stimuli

A total of 18 conventional poems were used as stimuli, of which one half was iambic and the other half trochaic. The original poems were manipulated by substituting single syllables with a “tack”-syllable. “Tacks” were placed at random positions and occurred in random number within a line. Except for one poem (D1, line two), lines which included tacks started earliest at the third verse. They could represent single syllabled words (“tack”) as well as multiple syllabled words (e.g., “tacktack”). The decision to use “tack” as the substituting syllable was based on two factors: (1) its percussive characteristic, for it is often used in music rehearsals to illustrate a piece’s rhythm, and (2) because it is the second syllable of “ticktack” (tick-tock), a word which commonly denotes a ticking sound, often used to illustrate, e.g., the ticking of a clock and thus suitable for temporal assignment.

### 2.3. Questionnaires

In an attempt to investigate possible relations to reading habits or to musical proficiency, further data was collected. *Reading habit questionnaire*: A questionnaire was developed to measure reading habits and their potential correlation with the overall reading performance of individuals. The following data were collected: Demographic data (age, gender, educational level), reading habits I (categories, e.g., newspaper, novels, etc.), reading habits II (percentage of reading/writing, analog vs. digital, and reading time spend with category), reading habits III (actual familial reading habits and during childhood, reading to other people privately/professionally), speaking/writing development, speaking habits, L2 languages, language (therapy) experiences, speaking anomalies (e.g., mumbling), and auditive habits (volume setting tendencies, i.e., loud/silent, music listening preferences). *MusA*. A short Questionnaire to Assess Musical Activity (Fernholz et al., 2018), which investigates music preferences as well as musical activity. After analyzing the two questionnaires for possible correlations between musicality and reading habits first, only factor *musical* was integrated in later analysis.

### 2.4. Recordings

For audio recordings, we used Sennheiser headset PC 8 USB with a frequency response of 42–17.000 Hz and Praat recording software (version 6.0.41; Boersma, 2001). Also, video-recordings (full body) were carried out with Sony video camera DCR-SR72.

### 2.5. Procedure

The study was conducted in the lab of the Center for Cognitive Science at the University of Freiburg. The experimental session started with reading a short info sheet about the procedure of the experiment. Next, participants had to fill out the questionnaires, which roughly took 10 min to complete. Participants then were instructed to place themselves in an upright position in front of the video camera. To ensure a fixed body position, participants had to locate both their forefeet close to a Gaffa tape line that was glued to the floor. After that, the experimenter positioned the headset on the participant’s head and made sure that the microphone was placed properly, with a maximum distance of 2 cm to the participants’ lips. Then the height of the video camera was adjusted, and image capture was focused. The last part of the set-up was a quick mic and recording test. Finally, stimuli-texts were handed over to the participant, with graphics turned upside-down to make sure that no time was given to reflect on genre ahead of reading. Before the reading of the stimuli was recorded, participants were asked to read aloud and according to one out of three conditions, *rhythmically*, to read in line with a beat (*tactus*), or *no instruction*. Then the Praat recording button was pressed and subjects were ordered to turn around the stimuli sheets and to start read. On average, recordings took roughly 10-15 min.

### 2.6. Data analysis

In a first step, a syllable table was prepared listing all syllable tokens chronologically for each poem, such that each row represented one syllable token. Further information for each syllable was assigned, e.g., the poem and the word the syllable belongs to, and the numeric index of a syllable in a word, line, and poem, etc. In another step, a subset from the recorded audio files was chosen, namely the poems “Im Grase” by Justinus Kerner *(A1*, 273 syllables), “Die Gunst des Augenblicks” by Friedrich Schiller (*D2*, 270 syllables), “Reiselied” by Hugo von Hofmannsthal (*C2* with 76 syllables; partly catalectic), “Mittag” by Theodor Fontane (*D1*, 68 syllables), “Das Sonett” by Johann Wolfgang von Goethe (*E1*, 154 syllables) and “Herbst” by Theodor Storm (*F2*, 150 syllables; this stimulus had been reduced by omitting stanzas 6 and 7). Stimuli *A1*, *D1*, *E1* were categorized iambic and *C2*, *D2*, and *F2* trochaic. For this reduced subset, all recorded sound files were separated per poem using Praat Software (version 6.0.53; Boersma and Weenink, 2018) and saved in .*wav* format. Then, for each .*wav* file a separate .*txt* format file with the corresponding stimuli text was generated, per poem and per participant. Next, we obtained automatically annotated Praat TextGrids with words and phones segmented and labeled, by applying WebMAUS Basic from BAS Web Services Version 3.1 (Schiel, 1999; Kisler et al., 2017) and by running the .*wav* and .*txt* files pairwise, as instructed. Using again Praat Software, the resulting TextGrids were inspected. If positioning of a boundary or an interval was imprecise, annotation of initial or final phones of the word was manually corrected. We added another tier for the boundaries for syllables. After annotation for all TextGrids was completed, praat scripts for duration and intensity (Reetz, 2020) were applied to perform computations for all labeled intervals to obtain the necessary data points for analysis. For duration, we used the output provided by the script for the variables for file index, label, i.e., either phon, syllable, word or line, corresponding to the annotation described above, and, starting points for each as well as duration for each. For intensity, the scripts’ output used were the values for mean-dB.

All resulting files were read into R. Additionally, the questionnaire data were transferred to Excel and also read into R. The data frames for syllable durations and intensities were then joined with the syllable table and with a subset of the merged questionnaire data. Four variables were added: (*i*.) *meter*, i.e., whether a poem was categorized iambic or trochaic, (*ii*) *stress*, meaning the predicted stressing of syllables according to the metrical grid. Two poets coded this variable by annotating *s* for strong and *w* for weak syllables, which led to almost strictly binary patterns with only a few exceptions per poem. Additionally (*iii*), *instruction*, encoding whether the poems should be read rhythmically (*rhythmic*), in line with a - individually induced and projected - beat (*on beat*, “im Takt”), or whether no instruction was given at all (*no instruction*). Next (iv.) the variable *musical* was derived from the answers given for questions 4 and 8 of the MusA questionnaire. First, participants were asked whether or not they had ever been musically active in their lives, i.e., by playing an instrument or by singing, and if so at what age and how many hours per week. If they answered “no,” they were annotated as not active. Otherwise, subjects were then asked: *In the last 12 months*, *how often have you been musically active?* One of following answer options could be checked for a) instrument as well as for b) singing: *Not at all*, *once a month or less*, *2–3 times a month*, *1 time per week*, *2–3 times per week*, *4–6 times per week*, *daily*. Answers of both parts (instrument + singing) were combined, i.e., when at least one answer in both parts was different from *Not at all*, the participant was coded as musical: *active*, otherwise *not active*. This resulted in six participants being categorized as *active*, and seven participants as *not active*.

From here on, all statistical analyzes were performed using the software R (version 4.1.1; R Core Team, 2020). We calculated mixed-effects regression models using the lmer function from the lme4 package (version 1.1–27.1; Bates et al., 2015). For all calculations, including nPVIs (adapted from http://cspeech.ucd.ie/Fred/nPVI.php), we used function *contrSum* for sum-coding from the car package (Fox and Weisberg, 2019). In sum-coding, the intercept represents the grand mean. Hence, the estimate difference between two conditions of a binary factor is two the times the estimate *β*. For estimating the *p*-values we used the function tab_model from the sjplot package (R package version 2.8.12, Lüdecke, 2022), using method *Satterthwaite* for estimating the degrees of freedom. Cohen’s *d* was estimated using the function lmd.score from the EMAtools package (version 0.1.4, Kleiman, 2021). We report Cohen’s d > 0.1.

We analyzed the data on two distinct levels, the syllable level (1) and the line level (2). All data, were the SOI exceeded 2000 ms, were eliminated. The data set yielded an average of 981 syllables per participant and, overall, 120 lines per participant.

#### 2.6.1. Syllable level

Level 1 investigated rhythmic signatures at the syllable level. Two subsets were created, one including only regular syllables and the other including only tack-syllables. The reason the data set was split this way was to generate a clean baseline for regular syllables so that the variance of the data patterns of the tacks could be compared to it.

For each response variable, two identical models were fitted. The first model fit was used to identify outliers in the residuals using the function boxplot() with range 1.5: only data where the residual did not exceed 1.5 times the inter-quartile range from the box were included in the data set that entered the second model fit, which is the one reported here.

##### 2.6.1.1. Syllable onset interval

*Duration* was sub-leveled into *syllable duration* (syllable onset to offset) and duration of the SOI, i.e., the beginning of the onset of a syllable until the onset of the succeeding syllable. We only report SOI, because this measure integrates speaking pauses (rests) and thus reflects rhythmicity better than the mere syllable duration. Pauses at the end of each line were excluded. Thus, in the SOI model fit for regular syllables, the dependent variable was *SOI*, the fixed effects predictors used were *stress*, *musical* and *meter*. *Participant* and *syllable* were included as random factors. For participant, the random intercept and the random slope for *stress* were included. Only the random intercept was included for *syllable*.

The structure of the model for tacks was almost the same, except that the variable for the index number of a tack within a line (*tack index*) was added as fixed effect predictor, and logically, *syllable* as random intercept was excluded for this model fit.

##### 2.6.1.2. Mean intensity

We used the same model fit described above, but the dependent variable was changed to mean intensity (*i_mean*).

#### 2.6.2. Line level

Level 2 examined rhythmic signatures at the line level. Specifically, we were interested in the rhythmic contrasts between adjacent syllables. These could be based on the durations or intensities of syllables, which both could be indicating stress.

Grabe and Low (2002) had introduced the nPVI as a measure of the variability of successive syllabic durations, in their work this was based on vowel length. In an modified adaption, Patel and Daniele (2003) had used it to compare French and English speech rhythms with rhythms in respective musical compositions. Here, we used the normalized nPVI index as an indicator for rhythmic variation within lines, based on the version presented by Cummins (n.d.)^2^, which calculates the nPVI for a sequence of syllable onsets:


$$nPVI=100\left[\overset{m-1}{\underset{k=1}{\sum }}\left|\frac{d_{k}-d_{k+1}}{\left(d_{k}-d_{k+1}\right)/2}\right|/\left(m-1\right)\right]$$

Next, an aggregated data frame for lines was produced from the syllable data frame, using the nPVI as the aggregation function over syllable SOIs (*npvi_soi*) and intensities (*npvi_i_mean*) for each line. We added the running line number for each poem (*line*), and the maximum number of syllables per line (*n_sylls*). This dataframe also contained the binary variable *tack line* indicating whether a “tack” was in a line or not, as well as the numerical variable *tacks per line* representing the number of tacks within a line. Furthermore, the binary variable *musical*, i.e., whether the participant was musically active or not, was added. For the analysis, the numeric variables *tack*, *line*, and *n_sylls* where centered, using the scale-function from the Base-R package, respectively named *tack_C*, *line_c*, and *n_sylls_C*. The variables *meter*, *musical*, and *instruction* where coded as factors.

For the line level, we will only report general model fits, which means that no distinct subsets for tack-lines and non-tack-lines were used for the data analysis. Instead, we introduced the variable *tack line* to indicate whether at least one tack syllable was present in the line.

#### 2.6.3. Normalized pairwise variability index for syllable onset intervals

For the dependent variable (*npvi_soi*) two identical models were fitted. The predictor models consisted of the fixed effects predictors *tack line*, *musical* and *meter*. The variables *participant* and *poem* were included as random intercepts. In addition to the random intercept for participant, the random slope for *tack line* was included. Again, we first identified outliers in the residuals using the function boxplot() with range 1.5. In the successive model fit, only data where the residual did not exceed 1.5 times the inter-quartile range from the box were included in the data set. We only report the second model fit here. In a second version of this model the binary variable *tack line* was replaced by the continuous variable *tacks per line* as a fixed effect predictor. Accordingly, in addition to the random intercept for participant, the random slope for *tacks per line* was included.

#### 2.6.4. Normalized pairwise variability index for intensities

The procedure for fitting the model was the same as just described for nPVI duration, except that the dependent variable was exchanged, i.e., *npvi_i_mean* was inserted instead.

Please note that in the further course, we will use the term “inactive” for the “not-active” level of the between-subject factor “musical” to ensure improved readability of the text.

## 3. Results

### 3.1. Syllable level

#### 3.1.1. Syllable onset interval

**Figure 1:**
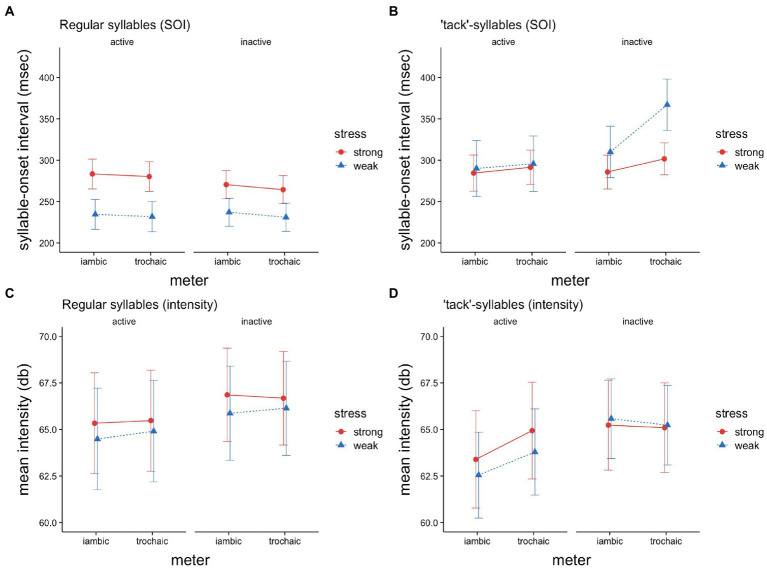
Syllable-onset intervals **(A,B)** and syllable intensities **(C,D)** for *regular syllables* **(A,C)** and ‘*tack’-syllables* **(B,D)**, as a function of stress (strong vs. weak), meter (iambic vs. trochaic), and musical activity (active vs. not active). The whiskers depict 95% confidence intervals.

The model fit for SOIs for regular syllables (Table 1 and see Figure 1A) revealed a significant main effect for *stress* (*p* < 0.001), i.e., on average the SOI for a strong syllable was spoken 41.08 ms (≅2×β, *Cohen’s* |*d*| = 4.77) longer than for a weak syllable. A significant two-way interaction with *musical* was found for *stress* (*p* = 0.025), meaning that for musically active readers, the average SOI for a strong syllable was an additional 7.26 ms (|*d*| = 1.58) longer. The model also revealed a significant main effect for *meter* (*p* = 0.013), indicating that SOIs in poems with iambic patterns were on average 4.26 ms longer, meaning that iambic poems were on average read slower than trochaic poems.

**Table 1:** Syllable onset intervals and mean intensity for regular syllables.

*Predictors*	SOI for regular syllables					Mean intensity for regular syllables				
	*β*	*ste*	*df*	*t*	*p*	*β*	*ste*	*df*	*t*	*p*
Intercept	254.16	6.77	31.81	37.52	**<0**.**001**	65.72	0.94	11.39	69.86	**<0**.**001**
Stress [strong]	20.54	1.71	23.98	11.99	**<0**.**001**	0.37	0.12	16.53	3.09	**0**.**007**
Musical [active]	3.31	5.63	15.30	0.59	0.566	−0.67	0.93	11.00	−0.72	0.489
Meter [iambic]	2.13	0.86	26969.86	2.48	**0**.**013**	−0.08	0.05	7876.23	−1.78	0.075
Stress [strong] * musical [active]	3.63	1.39	10.37	2.61	**0**.**025**	−0.01	0.11	10.97	−0.13	0.896
Stress [strong] * meter [iambic]	−0.12	0.85	27020.09	−0.14	0.890	0.09	0.04	7960.20	2.03	**0**.**043**
Musical [active] * meter [iambic]	−0.70	0.51	30000.07	−1.35	0.176	−0.06	0.03	9842.80	−2.03	**0**.**042**
Stress [strong] * musical [active] * meter [iambic]	0.06	0.51	30019.13	0.11	0.911	−0.02	0.03	9843.20	−0.74	0.458
Random effects										
σ^2^	2623.56					8.31				
τ_00_	5982.17 _corrected.syllable_					6.29 _corrected.syllable_				
	406.63 _vpnum.x_					11.23 _vpnum.x_				
τ_11_	21.53 _vpnum.x.stress [S.strong]_					0.14 _vpnum.x.stress [S.strong]_				
ρ_01_	0.81 _vpnum.x_					−0.02 _vpnum.x_				
ICC	0.71					0.68				
N	13 _vpnum.x_					13 _vpnum.x_				
	431 _corrected.syllable_					431 _corrected.syllable_				
Observations	10,024					10,306				
Marginal R^2^/Conditional R^2^	0.046/0.723					0.023/0.687				
AIC	109016.742					52376.487				

Bold values indicate statistical significance.

For tack syllables, *stress* appeared to have an effect, but the effect was not significant (*p* = 0.079). Surprisingly, speaking time for SOIs for weak tack syllables was on average 19.7 ms longer than for strong tack-syllables (|*d*| = 0.51). The analysis (Table 2) revealed a significant main effect for *meter* (*p* = 0.001), i.e., SOIs were 29.22 ms (|*d*| = 0.15) longer in poems with trochaic patterning. A main effect was also found for the *tack index* (*p* < 0.001), i.e., the index for the particular “tack” within a line. It shows that, on average, each increment of a tack syllable within a line increases the SOI by 15.84 ms (|*d*| = 0.18). In addition, the model yielded a significant two-way-interaction for *musical* with *meter* (*p* = 0.003), indicating that musically active subjects’ SOIs were on average 26.02 ms longer in iambic poems than in trochaic (|*d*| = 0.13). Although no main effect was found for *musical*, the analysis suggests a two-way-interaction of *stress* and *musical* (*p* = 0.066, |*d*| = 0.53), which is based on a three-way interaction with *meter* (*p* = 0.007). Figure 1B illustrates this result. While musically active readers seem to make an almost negligible distinction between strongly and weakly stressed tack-syllables, regardless of meter, musically inactive readers show a different pattern. In both iambic and trochaic poems, they exhibit longer SOIs for the weakly stressed syllables, an effect that is more pronounced in trochaic poems.

**Table 2:** Syllable onset intervals and mean intensity for variable *tack* syllables.

*Predictors*	SOI for tacks					Mean intensity for tacks				
	*β*	*ste*	*df*	*t*	*p*	*β*	*ste*	*df*	*t*	*p*
Intercept	286.85	9.46	37.04	30.32	**<0**.**001**	66.57	0.86	11.67	77.70	**<0**.**001**
Stress [strong]	−9.85	5.53	72.98	−1.78	0.079	0.66	0.17	131.27	3.79	**<0**.**001**
Musical [active]	−3.14	9.46	37.04	−0.33	0.742	0.36	0.86	11.67	0.42	0.683
Meter [iambic]	−14.61	4.40	5806.16	−3.32	**0**.**001**	−0.17	0.16	2062.01	−1.03	0.303
Tack index	7.92	1.95	5811.09	4.06	**<0**.**001**	−1.01	0.07	2061.68	−14.13	**<0**.**001**
Stress [strong] * musical [active]	10.33	5.53	72.98	1.87	0.066	0.28	0.17	131.27	1.60	0.112
Stress [strong] * meter [iambic]	−2.19	4.40	5805.03	−0.50	0.619	−0.21	0.16	2061.31	−1.31	0.189
Musical [active] * meter [iambic]	13.01	4.40	5806.16	2.96	**0**.**003**	−0.34	0.16	2062.01	−2.08	**0**.**038**
Stress [strong] * tack index	−1.25	1.95	5812.40	−0.64	0.521	−0.23	0.07	2061.89	−3.18	**0**.**002**
Musical [active] * tack index	−4.70	1.95	5811.09	−2.41	**0**.**016**	−0.56	0.07	2061.68	−7.86	**<0**.**001**
Meter [iambic] * tack index	1.89	1.95	5807.74	0.97	0.333	−0.06	0.07	2061.80	−0.82	0.414
Stress [strong] * musical [active] * meter [iambic]	−11.86	4.40	5805.03	−2.69	**0**.**007**	−0.24	0.16	2061.31	−1.46	0.145
Stress [strong] * musical [active] * tack index	−0.18	1.95	5812.40	−0.09	0.928	0.01	0.07	2061.89	0.20	0.839
Stress [strong] * meter [iambic] * tack index	3.47	1.95	5806.66	1.78	0.075	0.07	0.07	2061.17	1.00	0.320
Musical [active] * meter [iambic] * tack index	−2.64	1.95	5807.74	−1.36	0.175	−0.04	0.07	2061.80	−0.49	0.623
Stress [strong] * musical [active] * meter [iambic] * tack index	3.17	1.95	5806.66	1.62	0.104	0.11	0.07	2061.17	1.51	0.132
Random effects										
σ^2^	6803.36					9.26				
τ_00_	905.91 _vpnum.x_					9.15 _vpnum.x_				
τ_11_	144.96 _vpnum.x.stress [S.strong]_					0.05 _vpnum.x.stress [S.strong]_				
ρ_01_	−0.72 _vpnum.x_					0.80 _vpnum.x_				
ICC	0.13					0.50				
N	13 _vpnum.x_					13 _vpnum.x_				
Observations	2059					2099				
Marginal R^2^/Conditional R^2^	0.100/0.220					0.108/0.552				
AIC	24033.687					10766.851				

Bold values indicate statistical significance.

**Figure 2:**
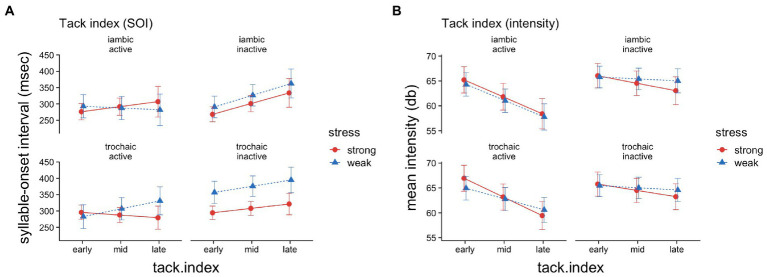
Syllable-onset intervals **(A)** and syllable intensities **(B)** for *tack* syllables, as a function of stress (strong vs. weak), meter (iambic vs. trochaic), musical activity (active vs. not active), and tack index (small ~0, mid, large ~5). The whiskers depict 95% confidence intervals.

On top of the main effect found for *tack index*, the model shows a reliable two-way interaction of *musical* and *tack index* (*p* = 0.016), as shown in Figure 2A. This indicates that the positive main effect for *tack index* was mainly due to the musically inactive readers, where each additional tack in a line increased the main effect of SOI by 4.7 ms, while for musically active participants it was decreased by the same amount.

In the analysis, *stress* appeared to interact with *meter* and *tack index*, however, the effect turned out not to be significant (*p* = 0.075). Also, no two-way-interactions were found for *stress* and *meter* or for *stress* and *tack index*.

As the graphical representation suggests (Figure 2A), the overall results are due to a specific pattern: For musically inactive readers the SOIs tend to be longer overall for the tack syllables coded “weak” compared to the tack syllables coded “strong,” independent of meter. Musically active readers, on the other hand, show a different pattern for iambic vs. trochaic. Here, we find shorter SOIs for the weakly stressed tacks in iambic poems compared to the strongly stressed ones found with a larger tack index, and vice versa, longer SOIs with a larger tack index for the weakly stressed tacks compared to the strongly stressed ones in trochaic poems. As the graph also illustrates, the pattern for trochaic poems for musically active readers is particularly interesting, as SOIs for weak syllables are shorter than for strong syllables with a smaller tack index, but with a larger tack index the pattern reverses, and SOIs for strong syllables are even shorter than with the smaller tack index, and SOIs for weak syllables become noticeably longer. To analyze whether the effect of tack index with regard to the specific pattern for musically active readers, is statistically tenable, we additionally computed a model for musically active readers only. The model revealed a significant three-way-interaction of *stress*, *meter*, and *tack index* (*t* = 2.673, *p* = 0.008).

#### 3.1.2. Mean intensity

For regular syllables, the analysis (Table 1) reveals a significant effect for *stress* (*p* = 0.007). As shown in Figure 1C, the pronunciation of stressed syllables was on average 0.74 dB (|*d*| = 1.52) louder than that of weak syllables. The model suggests an effect for *meter*, i.e., the mean intensity was increased by about 0.16 dB in trochaic poems, however, the effect was not significant (*p* = 0.075). The model also yielded a significant two-way-interaction between *stress* and *meter* (*p* = 0.043), indicating that in iambic poems, strong syllables were spoken 0.18 dB louder than weak syllables. Noticeably, the mean intensity differs less between strong vs. weak syllables in trochaic poems. Although no main effect for *musical* (|*d*| = 0.43) was found, there is a significant two-way-interaction of *musical* with *meter* (*p* = 0.042), showing that musically active readers read less intensively overall in iambic compared to trochaic poems.

The analysis for tacks (Table 2) shows a main effect for variable *stress* (*p < 0*.001), indicating that stressed tack-syllables were overall about 1.32 dB (|*d*| = 0.66) more pronounced. Figure 1D illustrates that this effect was established by a particular pattern: mean intensities for musically active readers were elevated for stressed syllables compared to unstressed syllables and, notably, correspondingly for both iambic and trochaic poems. Conversely, mean intensities for musically inactive readers show no contrast between stressed and unstressed syllables, and likewise, for both meters. The model also yielded a main effect for *tack index* (*p* < 0.001), showing that each increment of a tack syllable within a line decreases intensity by 2.02 dB (|*d*| = 0.62). Furthermore, we found a significant two-way interaction of *stress* with *tack index (p = 0*.002, |*d*| = 0.14*)*, such that the effect was even more pronounced for strong syllables, with an additional average decrease of 0.46 dB (see Figure 2B). As can also be seen in the graph, although no main effect was found for *musical* (|*d*| = 0.25), there was a significant two-way-interaction with *tack index* (*p* < 0.001, |*d*| = 0.35), i.e., the more tacks occurred earlier in the line, the higher the overall mean intensities for musically inactive readers. Interestingly, the model also yielded a significant two-way-interaction between *musical* and *meter* (*p* = 0.038), revealing that with a higher tack index, mean intensities decreased by about 0.68 dB on average for musically active readers and for iambic poems.

### 3.2. Line level

#### 3.2.1. Normalized pairwise variability index for syllable onset intervals

**Figure 3:**
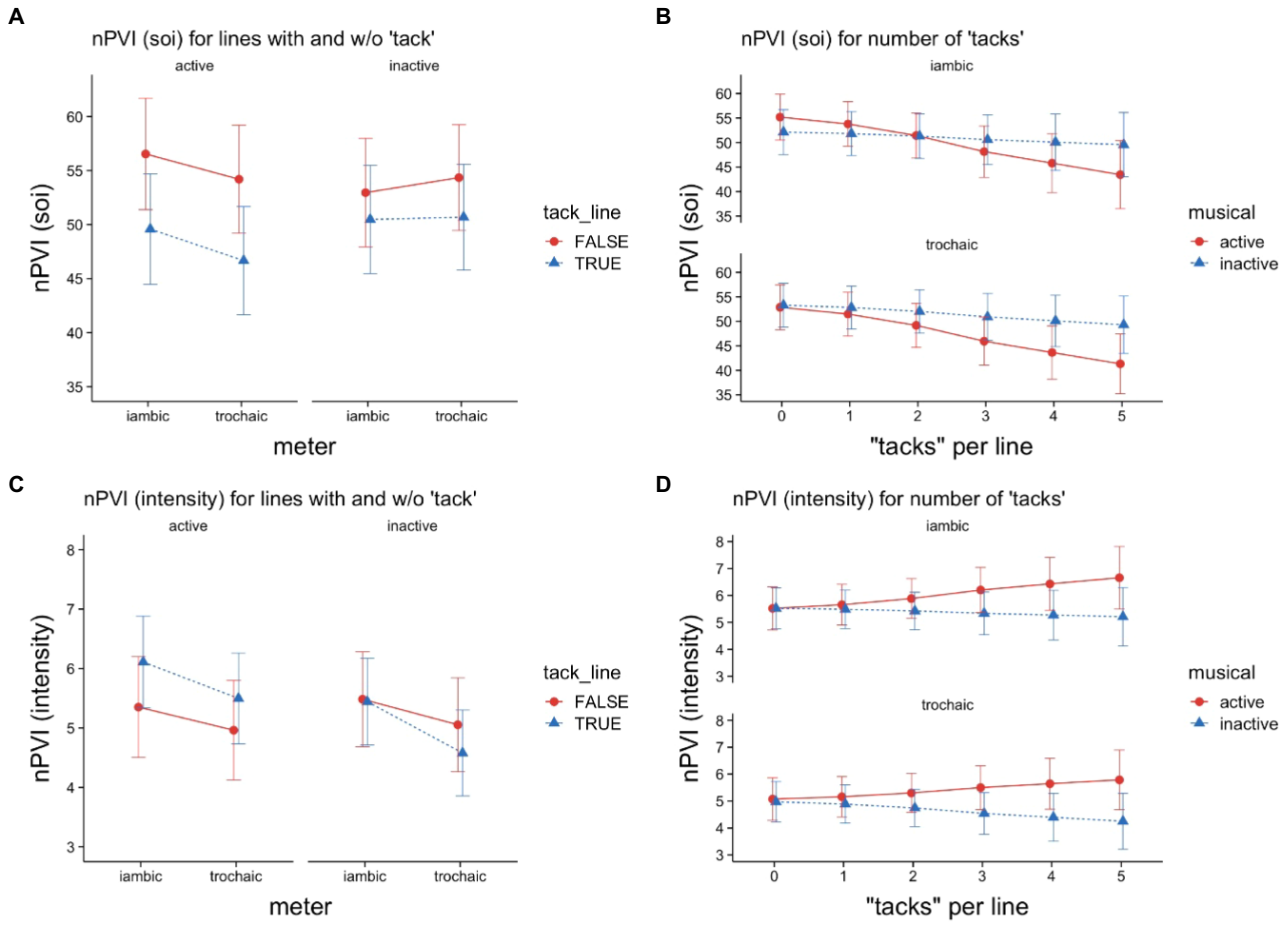
Illustration of analyses on the line level. *SOI-based nPVIs* **(A,C)** and *intensity-based nPVIs* **(B,D)**, as a function of meter (iambic vs. trochaic), musical activity (active vs. not active), and tack line [whether or not one or more tacks are present in a line **(A,B)**], or tacks per line (the number of tacks in a line), ranging from low = 0 to high = 5 **(B,D)**. The whiskers depict 95% confidence intervals.

The analysis (Table 3) of the nPVI for SOI for lines with tacks versus lines without tacks revealed a significant main effect of *tack in line* (*p* < 0.001). It shows that the nPVI values as an indicator of rhythmic contrast for SOIs were different for lines with and without tacks, with lines without tacks having the higher value (*β* = 2.58, |*d*| = 0.47) compared to lines with tacks. Thus, as detailed in Figure 3A, the nPVI for SOIs for lines without tacks was more pronounced for both iambic and trochaic conceptualized poems than for lines that contained tacks. Additionally, a significant two-way interaction of *tack in line* with *musical* was found (*β* = 1.04, *p* = 0.015, |*d*| = 0.19). Figure 3A also illustrates that, surprisingly, the nPVI for SOIs for lines with tacks is significantly reduced for musically active readers compared to lines without tacks. On the other hand, the nPVI for SOIs for musically inactive readers does not seem to differ much for lines with or without tacks. Although there was no main effect for *musical*, the model also yielded a two-way interaction with *meter* (*β* = 0.86, *p* = 0.045, |*d*| = 0.10).

**Table 3:** Combined table for nPVI SOI and nPVI mean intensity for lines with and without tacks.

*Predictors*	nPVI syllable onset duration (SOI)					nPVI mean intensity				
	*β*	*ste*	*df*	*t*	*p*	*β*	*ste*	*df*	*t*	*p*
Intercept	51.93	1.65	4.65	31.52	**<0**.**001**	5.31	0.25	13.90	21.06	**<0**.**001**
Tack in line [F]	2.58	0.43	647.92	6.01	**<0**.**001**	−0.10	0.11	11.03	−0.88	0.398
Musical [active]	−0.18	0.61	11.12	−0.30	0.768	0.17	0.21	11.01	0.81	0.433
Meter [iambic]	0.46	1.59	4.06	0.29	0.788	0.29	0.15	4.13	1.93	0.124
Tack in line [F] * musical [active]	1.04	0.43	647.28	2.43	**0**.**015**	−0.23	0.11	11.03	−2.05	0.065
Tack in line [F] * meter [iambic]	−0.22	0.43	1537.34	−0.51	0.610	−0.08	0.05	1524.42	−1.80	0.072
Musical [active] * meter [iambic]	0.86	0.43	1537.09	2.00	**0**.**045**	−0.04	0.05	1524.17	−0.80	0.425
Tack in line [F] * musical [active] * meter [iambic]	0.07	0.43	1537.15	0.17	0.864	0.03	0.05	1524.17	0.58	0.564
Random effects										
σ^2^	281.64					3.22				
τ_00_	2.44 _participant_					0.54 _participant_				
	13.72 _poem_					0.12 _poem_				
τ_11_	0.01 _participant.tack_line [S.FALSE]_					0.13 _participant.tack_line [S.FALSE]_				
ρ_01_	1.00 _participant_					0.18 _participant_				
ICC						0.20				
N	13 _participant_					13 _participant_				
	6 _poem_					6 _poem_				
Observations	1,560					1,558				
Marginal R^2^/Conditional R^2^	0.029/NA					0.042/0.230				
AIC	13260.898					6354.937				

Bold values indicate statistical significance.

Further analysis explored the possibility that the number of tacks within a line might play a modulating role. As highlighted in Table 4, the model showed a significant main effect for the variable *tacks per line* (*β* = −1.49, *p* < 0.001, |*d*| = 2.87), i.e., with each additional tack more in a line, the nPVI-value decreased by 2.98. In addition, the analysis revealed a two-way interaction of *tacks per line* with *musical* (*β* = −0.84, *p* = 0.016, |*d*| = 1.62), meaning that the effect was even stronger for musically active readers, with an additional average decrease of 1.68. *Musical* appeared to interact with *meter*, but the effect turned out to be not significant (*β* = 0.79, *p* = 0.065). In Figure 3C this is graphically detailed, such that for iambic and trochaic meters, the nPVI for the SOI decreased for musically active readers with a higher number of tacks within a line. However, the graph also shows that this effect was much less pronounced for musically inactive readers in the trochaic poems and almost negligible in the iambic poems.

**Table 4:** Combined table for nPVI mean intensity and nPVI SOI for tack count.

*Predictors*	nPVI syllable onset duration (SOI)					nPVI mean intensity				
	*β*	*ste*	*df*	*t*	*p*	*β*	*ste*	*df*	*t*	*p*
Intercept	51.88	1.55	4.76	33.52	**<0**.**001**	5.31	0.25	13.86	20.93	**<0**.**001**
Tacks per line	−1.49	0.30	12.17	−5.01	**<0**.**001**	0.04	0.08	11.15	0.53	0.609
Musical [active]	−0.18	0.61	11.12	−0.29	0.777	0.17	0.21	11.01	0.80	0.440
Meter [iambic]	0.36	1.49	4.08	0.24	0.820	0.29	0.15	4.12	1.93	0.124
Tacks per line * musical [active]	−0.84	0.30	12.02	−2.81	**0**.**016**	0.14	0.08	11.13	1.87	0.088
Tacks per line * meter [iambic]	0.06	0.28	1528.43	0.22	0.829	0.04	0.03	1526.47	1.39	0.164
Musical [active] * meter [iambic]	0.79	0.43	1526.24	1.85	0.065	−0.02	0.05	1524.15	−0.55	0.585
Tacks per line * musical [active] * meter [iambic]	−0.08	0.28	1526.28	−0.29	0.770	0.00	0.03	1524.18	0.02	0.984
Random effects										
σ^2^	282.25					3.20				
τ_00_	2.43 _participant_					0.54 _participant_				
	11.81 _poem_					0.12 _poem_				
τ_11_	0.13 _participant.tacks_per_line_					0.07 _participant.tacks_per_line_				
ρ_01_	−0.02 _participant_					−0.17 _participant_				
ICC	0.05					0.20				
N	13 _participant_					13 _participant_				
	6 _poem_					6 _poem_				
Observations	1,560					1,558				
Marginal R^2^/Conditional R^2^	0.025/0.073					0.040/0.236				
AIC	13268.573					6353.326				

Bold values indicate statistical significance.

#### 3.2.2. Normalized pairwise variability index for intensities

For nPVI mean intensity for lines with tacks versus lines without tacks, no main effect was found for *tack in line* (see Table 3), and although the model suggests that *tack in line* interacted with musical, the effect turned out to be not significant (*β* = −0.23, *p* = 0.065, |*d*| = 1.23). Additionally, *tack in line* appeared to interact with *meter*, but the effect was also not significant (*β* = −0.08, *p* = 0.072). Also, there was no main effect for meter. The graphical inspection (see Figure 3B) of this result shows that the nPVI for mean intensity was higher for musically active readers in both iambic and trochaic poems when the lines contained tacks. In contrast, the nPVI for mean intensity for musically inactive readers decreased for lines with tacks compared to “normal” lines.

No main effect was found for the number of tacks within a line (see Table 4). Again, the model suggests *tacks per line* to interact with *musical*, however, the effect turned out to be not significant (*β* = 0.14, *p* = 0.088, |*d*| = 1.12). Figure 3D illustrates this result, with both musically active and musically inactive readers showing a comparably high nPVI for the mean intensity at a lower number of tacks per line (<2). However, this pattern changes as the number of tacks within a line increases, for both iambic and trochaic poems. Then we find that the nPVI for mean intensity tends to be higher overall for musically active readers than for musically inactive readers.

#### 3.2.3. Instruction

**Figure 4:**
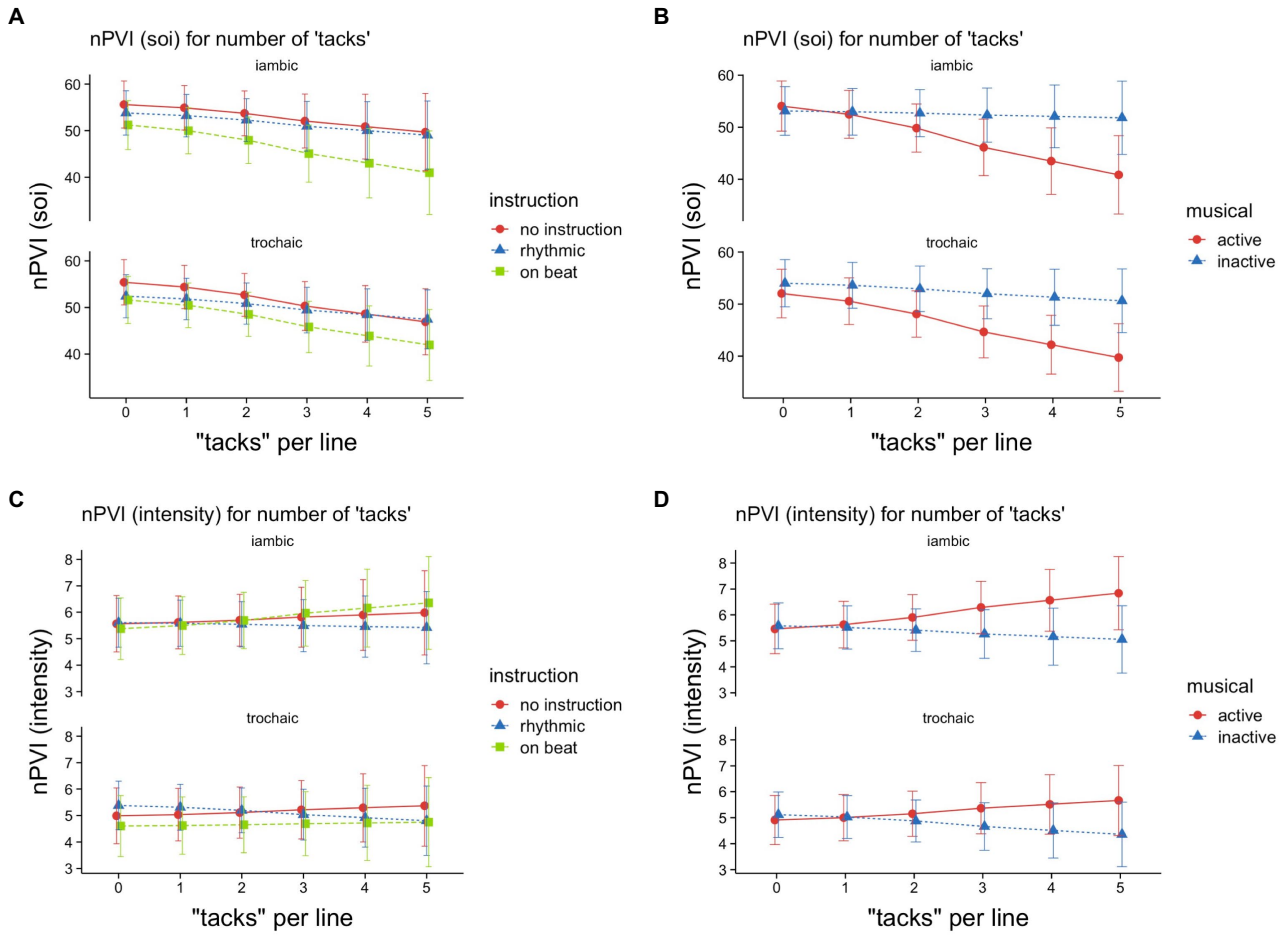
Illustration of the analysis of variable *instruction* on the line level for two models: including variable *instruction* **(A,C)** vs. including the interaction of *instruction* and *musical* **(B,D)**. *SOI-based nPVIs* **(A,B)** and *intensity-based nPVIs* **(C,D)**, as a function of meter (iambic vs. trochaic), and either instruction (no instruction vs. rhythmic vs. on beat; **A,C**) or musical activity (active vs. not active; **B,D**), and tacks per line (the number of tacks in a line, ranging from low = 0 to high = 5; **B,D**). The whiskers depict 95% confidence.

Each participant was assigned to one of three instruction groups (*no instruction*, *rhythmic*, *on beat*). In a previous analysis, we found that the *instruction* was less effective than expected, so we did not include it as a predictor in our models. However, we found that musicality was confounded with our instruction groups: Three musically active but only one inactive participant received no instruction, three active and two inactive subjects were instructed to read “rhythmically,” while no active, but four inactives were instructed to read “on beat” (“im Takt”). Therefore, we fit two more models – one for SOI-based nPVIs and one for intensity based nPVIs – to reaffirm that the musicality effects were not due to the instruction assignment. In these models, we had both variables – *musical* and *instruction* – interact with the same predictors, so any effect of musicality should disappear, or at least be reduced, if it can be attributed to instruction. While instruction did seem to account for some variability in the data (see Figures 4A,C), its inclusion in the model appeared to have virtually no effect on the general effect pattern of musicality, as can be seen in Figure 4B,D, when compared to the result of the simpler model (see Figure 3B,D): The significant interaction between *musical* and *tacks per line* for nPVI (SOI) remained significant (*β* = −1.04, *t* = −2.66, *p* = 0.008). Also, the main effect of *tacks per line* was unaltered (*β* = −1.55, t = −5.01, *p* < 0.001). We found one difference though: While there was no main effect of *musical* in the analysis above, the model now yields this main effect (*β* = −1.3, *t* = −2.12, *p* = 0.034), indicating that musically active participants read less rhythmically. However, since this effect only appears after the additional inclusion of the instruction variable, it is very likely that this effect is a suppressor effect due to the confounding of the two variables.

As for *instruction* itself: There appears to be an effect of *instruction*, as the nPVI (SOI) was significantly increased when no explicit instruction was given (*β* = 2.19, *t* = −2.82, *p* = 0.005). For intensities, the nPVI for poems in *iambic* meter was slightly reduced when participants were instructed to read rhythmically (*β* = 0.15, *t* = −2.36, *p* = 0.018).

However, as shown above, these effects did not substantially alter the general pattern of effects of *musical*. We therefor conclude that the omission of the instruction predictor was justified.

## 4. Discussion

Our goal was to investigate rhythmic patterns during oral reading of metrically regular, rhymed language (MRRL) and whether readers would realize a poem’s metric conceptualization, i.e., either as iambic or trochaic, by applying duration or intensity-based syllable patterning. Furthermore, we wanted to investigate whether the integration of the semantically meaningless syllable *tack* leads to interference. The aim of this was to gain insight into the extent to which readers can maintain a governing metrical grid during oral reading. Our idea was that musically inactive individuals would be more easily irritated by *tacks* than musically trained individuals. One premise for this would be that musically active individuals are more likely than musically inactive individuals to subordinate bottom-up processing in reading MRRL to a higher-level temporal relationship, i.e., top-down processing (compare Strait et al., 2010; Zhang et al., 2015). However, we also assumed that an increasing number of tacks in a verse would make it more difficult for both groups to maintain the main metrical grid.

At the syllable level, the results found for SOIs for the regular syllables show that both groups exhibited a stress pattern that corresponded to the underlying iambic or trochaic conception of the poems (cf. Figure 1A), with longer SOIs for the stressed syllable and shorter SOIs for the weak syllable. This result is in line with our simple stress hypothesis (H1). Remarkably, the musically active group showed a more pronounced difference between strong and weak syllables. This is in line with the OPERA-hypothesis (Patel, 2011, 2012), which assigns a higher temporal precision to musically active readers, and is thus supporting H4a.

For regular syllables, the strong-weak distinction was also replicated for intensity patterns (cf. Figure 1C), consistent with H1. The stress difference found was slightly stronger in the iambic conceptualized poems though, contrary to H2. According to the iambic-trochaic law (Hayes, 1985, 1995), the perception of an aural signal should be metrically biased, in that trochaic units are associated with intensity based stress marking, and iambic units with duration based marking. If perception preferences (but see Hay and Diehl, 2007) can be transferred to reading aloud, then we should have found a greater intensity difference for strongly and weakly stressed syllables in the trochaic poems. One explanation for this result could be that readers have to be more focused in iambic meter, which is less preferred in the everyday German. Also, contrary to our expectation that musical advantage also positively affects the use of intensity to mark stress (H4c), there was no difference between musically active and inactive readers. Overall, the results for regular syllables corroborate the findings by Breen (2018) and Breen and Fitzroy (2021), showing that both, duration and intensity, are used to realize the specific poetic sound quality and metrical discrimination during oral poetry reading.

We also specifically examined how “tacks” replacing regular syllables would affect SOIs and intensities. For SOIs the insertion of tacks in verse lines resulted in a different pattern compared to regular syllables, in that the weak syllables had longer SOIs (cf. Figure 1B). Interestingly, musically active readers seemed to exhibit no effect between strong and weak tack-syllables, regardless of meter. The SOIs for musically inactive readers, on the other hand, showed a clear difference for strongly and weakly stressed tacks, however with prolonged syllable reading times for the weakly stressed tack-syllables, as predicted by H7. This effect was more pronounced in trochaic poems than in iambic ones. Thus, our “leveling” hypothesis (H6), which expected that tacks are pronounced more similar to each other, leading to similar SOIs, independent of meter, seems to hold for musically active readers only, whereas musically inactive readers seem to experience overall more interference from “tacks”, resulting in lengthened SOIs of weakly stressed tack syllables. This suggests that tacks lead to a clash between top-down processing, i.e., the projection of the main metrical grid, and bottom-up processing, i.e., the assumption that tacks must be emphasized (H7). The interference is stronger in trochaic meter, where the weak syllable is the last syllable of the metrical unit. This result suggests that musically inactive readers chose to separate metrical units more strongly by pausing after a weakly stressed tack at the end of a trochaic unit. They thus appeared to have marked the boundaries of metrical units, instead of marking stress. This corresponds to H3, which was however not supported by the data for regular syllables. An alternative interpretation would be based on preference-hypothesis (Domahs et al., 2008; Wiese and Speyer, 2015), i.e., in German, a trochaic pattern is preferred, and musically inactive readers – in the absence of distinctive bottom-up information – may just locally fall back into a trochaic pattern. In other words, it could be that musically inactive readers superimpose the trochaic pattern preferred in everyday speech, even if they have globally derived the appropriate main metrical grid, e.g., iambus, and have applied it to tack-free verses before. In this case, SOIs would be still used to mark stress in iambic poems and musically inactive readers would switch to trochaic meter when they read tacks.

As illustrated (cf. Figure 2A), the SOI increased with tack index, but stronger so for musically inactive readers. For musically active readers, the tack index seems to have a complex effect on stressed and unstressed tacks. In iambic poems, the more tacks in a line, strongly stressed tack-syllables become lengthened even further, whereas weak tack-syllables become shorter with an increasing tack index. This result seems to support H10a. However, in trochaic poems, the opposite pattern emerged, which however did not amount to a significant four-way-interaction of *stress*, *musical*, *meter* and *tack index* (*p* = 0.10). Nevertheless, the three-way-interaction of stress, meter and tack index for musically active readers supports our interpretation.

The particular pattern found for musically active readers suggests that with an increasing number of tacks SOIs are used for marking the boundaries of the respective metrical unit, rather than to signal prominence for strong vs. weak tack-syllables. Thus, the iambic or trochaic stress pattern “encapsulated” in the metrical unit seems to prevail due to top-down processing of the metrically regular, rhymed language (H10).

In contrast, and interestingly so, for musically inactive readers the pattern of SOIs for tacks appears to be similar for both, iambic and trochaic poems, in that SOIs for strongly stressed tacks are shorter than for weakly stressed tacks. The consistent effect of stress suggests that metrical information is also maintained by inactive readers. The stress effect was however stronger in trochaic poems. While the tack index slowed down reading in general, it did not change this pattern of results. Therefore, the two suggested interpretations for the general effect of tacks – metrical unit vs. preference for trochaic – are valid independent of the number of tacks in a line.

In general, we find, in both groups, indicators for top-down-processing in reading tacks, however differently so. While musically active readers seem to use the “metric unit”-strategy for both, iambic and trochaic versions, musically inactive readers appear to use this strategy only in trochaic poems. In iambic poems they appear to fall back into trochaic meter, which is the dominant pattern in German.

Looking at mean intensities for tack-syllables, musically inactive readers show no difference between strong and weak tack syllables (*cf*. Figure 1D), whereas musically active readers clearly used intensity to mark prominence, as predicted in H5. The differential effect of musicality however supports H10c. Prominence marking by musically active readers was even more pronounced in trochaic poems, in support of H8.

The significant negative main effect of tack index on intensity means that tacks were generally spoken more quietly as the number of tacks increased (*cf*. Figure 2B). This could point at readers losing confidence about the metrical grid when more tacks occur in a line. For musically inactive readers, intensity decreased even more rapidly for strong syllables. Musically active readers, on the other hand, showed a different pattern for iambic and trochaic items. While they overall clearly used intensity for prominence marking, only in iambic poems did they do so for later tacks as well. This seems paradoxical, as prominence marking on tacks was overall stronger in trochaic poems, and hence seems to be easier here. However, as the corresponding results for SOIs clearly point toward the metrical unit hypothesis, where the lengthening of the unit-final syllables indicates grouping into metrical units, this effect may also be responsible for increasing intensities of unit-final syllables on later tacks (compare Wagner, 2022).

We also calculated the nPVI, aggregating neighboring syllable duration and intensity contrasts per line. The nPVI provides a simpler measure for calculating rhythmic contrasts (see H12+). Furthermore, the nPVI revealed additional properties of rhythmic processing, compared to the syllable level.

The nPVI over SOIs (*cf*. Figure 3A) revealed that oral MRRL-reading was rhythmically more pronounced in lines without tacks than in lines with at least one tack syllable. While this effect holds for both musically active and inactive readers, it was stronger for active readers. This nPVI pattern mirrors the syllable-based SOI effects of stress marking very well (*cf*. Figures 1A,B). These results clearly show that the lack of distinctive phonemic qualities in tacks disturbs the maintenance of the metric grid, which then solely depends on top-down projections.

Musically active readers also read the iambic lines more rhythmically than the trochaic lines, with or without tacks, while musically inactive readers did not show a meter effect. This pattern cannot be found in the syllable data. As trochaic patterns fit the general preference of German, this might have led musically active readers to smoother reading, as the meter is clearly identifiable. Iambic lines may require more stress distinction to be identifiable. Why we did not find this result on the syllable level, remains an open question.

Although musically active readers showed a quite steep decline of rhythmicity with high number of tacks in a line, the meter effect still prevailed (*cf*. Figure 3B). Inactives, however, showed a slower decline, and no signs of differentiation between meters whatsoever.

If tack-syllables were processed as nonsense-syllables, it might be possible that some syntactic form had been projected onto them. Thus, an alternative interpretation would hold that the effects found could be due to “surprisal” (Levy, 2008). Any results found could then have been driven by sentence processing and syntax-driven predictions related to timing. If we had replaced only a specific class of words with tacks, indeed, there could have been a learning effect, for example, if readers had realized that only nouns had been replaced. However, in our stimulus material, “tacks” substituted *different* word categories. Thus, although tacks remain unpredictable, they are likely to be perceived very quickly as placeholders, i.e., as words that fit any context. Furthermore, even if projections were occasionally possible, then presumably so only in verses including one, max. two tacks. It seems unlikely that readers were able to maintain a clear syntactic form in verses with more than two tacks, especially, since in poetry, grammatically correct syntax is often systematically broken or transformed. Therefore, we strongly assume that readers assigned less of a functionally relevant syntactic role to “tacks” during reading because of the poeticized language. Also, it is more plausible that with an increasing number of tacks within a line, syntactical processing as well as semantic comprehension become less important, whereas ‘keeping the rhythm’ while reading should become the main goal in order to complete the task at hand, which was to read the poem out loud.

However, another alternative and more likely interpretation would be that syntactic predictions, enabled by the meaningful regular syllables, may be a factor as important as phonetic structure for extracting, updating, and maintaining a leading metrical grid, because unlike the tack-syllables the regular syllables combine to form words and phrases. Thus, effects found for tack-syllables could also be explained by a tack induced weakening of the ongoing syntactic prediction process, disrupting the syntax-aligned metrical prediction of phrasal stress, as suggested by Hilton and Goldwater (2021). Thus, if there are fewer syntactic predictions possible, or non at all, because a line contains multiple meaningless tacks, then it is possible that there is less of a boost to metrical processing from these non-syntactic bottom-up cues, which in turn could have led readers’ performance to become more dependent on the top-down projection of the main metrical grid. For this, musicians might have overall more experience in keeping the beat while maintaining a meter and simulitaneously realizing a rhythm “in line” with it. This alternative interpretation is supported by the results found for the nPVI for SOIs, indicating that as the number of tacks per line increased (and thus as syntax within a line/stanza became more impoverished), there was less differentiation of meter.

For musical active readers we found a higher intensity-based nPVI for lines including tacks compared to lines without tacks. This result confirms the pattern found at the syllable level (cf. Figure 1D), and corroborates our interpretation, that musically active readers use intensity more for prominence marking in lines containing tacks. This would speak in favor of a more dominant role of top-down processing in musically active readers compared to inactives.

Although we expected for *intensity* that main effects found at the syllable level would also be visible at the line level, this was not the case. This was true for meter. On the one hand, the two-way interactions suggest that there may be a power problem. On the other hand, it would be possible that “tacks” represent syllable-like sounds which, due to their CV structure, provide little potential for intensive pronunciation anyway as well as for intensity variation: The syllable is overall short, but also the vowel is to be spoken short, and the ratio of voiceless vs. voiced phonemes is 3:1. Therefore, although a “tack” might be *perceived* as a ‘syllable’ which is to be stressed, it leaves little room for intensity variation in actual production. What is more, for the realization of stress and meter for tacks, it is also quite possible that pitch could be a better discrimination criterion for equal syllables, especially if ‘tacks’ follow each other immediately. In our study, the decision not to include pitch was based on a work by Zahner et al. (2019), who showed that although pitch contributes to the prominence, it must not necessarily add further information on the processing and vocalizing of metric information/projection. Nevertheless, a follow-up experiment should also investigate pitch (compare Breen and Fitzroy, 2021). However, the results at the syllable level show that intensity is indeed used for prominence marking, but rather by musically active readers than inactives. The syllable level thus seems to be better suited than the nPVI to investigate fine grained rhythmic and modulating (intensity) aspects of oral reading of poetry and the associated top-down and bottom-up processes.

Our findings support the notion that there is no ‘cut-off’ dichotomy between top-down and bottom-up processes (Rauss and Pourtois, 2013) during reading poetry aloud. In the context of the predictive model of music (Vuust et al., 2022) our results can indirectly contribute to the debate (e.g., active inference, Koelsch et al., 2019), since investigating SOIs and intensity in reciting poetry appears to be closely linked to prediction. At least for conventional poetry, the arrangement of the syllable sequences, respectively the composition of words within a line toward the stanza allows for a structured temporal distribution of their sounds. This in turn establishes a rhythm from which a “beat” can be induced, and against which a meter can be build up. Although the words and syllables of the poem are known from everyday speech (compare also Wagner, 2002, 2008), their pronunciation duration and accentuation are predicted top-down and subordinated to the selected model (e.g., choice of meter). With each stanza, reading can be further rhythmically adapted to it, i.e., ‘strengthening the metric model’ (Vuust et al., 2018). Specifically, for musically active readers, the patterns found in our study suggest that both SOIs and intensity are used to do so. Since musical training can improve and shape temporal precision (Danielsen et al., 2022) and metrical discrimination (Nave-Blodgett et al., 2021), it is reasonable to assume that musically active readers find it easier to quickly and accurately determine the leading meter of a poem. If their reading is oriented more toward the sound gestalt of the text and less toward comprehension, this should potentially minimize prediction errors regarding timing. The integration of multiple “tacks” however clearly led to distortions of the rhythmic reading pattern (i.e., higher prediction error) and the overall temporal distribution within the context of the line/stanza. This phenomenon shows the importance of the phonetic characteristics of a syllable for the rhythmic quality of a text. Especially in the context of reading out loud, the interaction of meter projection with the actual articulatory muscle production of the sound itself is fundamental for precise timing and hence related to predictive processes. Overall, musically active readers appeared to be better at adapting sensory input to the chosen prediction model, aka “resampling the evidence” (Vuust et al., 2018, p. 25). They presumably did so by “attenuating or suppressing precision of prediction errors” (*ibid*.) using the articulatory gestures, thus enabling sensomotoric synchronization, which, in turn, supports predictive coding.

### 4.1. Limitations

We coded the meter by the variable “stress” for each poem. However, there were few cases in a sequence of 3 or 4 tacks in which a tack was omitted during oral reading. For these, the manual Praat annotation could not assign which exact tack was omitted. Thus, for these few cases it could be possible that the assignment of the stress variables does not exactly match the tacks or their correct position during reading.

Another limitation of our study is that it employs only a small sample size of 17, of which 4 had to be excluded due to recording problems, leaving only 13 participants (6 musically active, 7 musically inactive) with usable data. Thus, for musicality as between-subject factor, one can criticize that a it does not provide reliable estimates. Some effects found turned out to be only marginally reliable. Hence, for these effects, evidence is inconclusive and further research is needed. However, the problem is somewhat mitigated by the fact that the amount of data points per subject is sufficiently high on the syllable level (on average, 981 syllables per participant) and with the aggregated version on the verse level (overall, 120 verse lines per participant).

In our analysis, we focused primarily on the post-hoc variable *musicality*. However, one aspect of the experimental set-up was the reading instruction (no instruction, rhythmic, on beat). Obviously, the variable *instruction* and the variable *musicality* were confounded. However, our post-hoc analysis including both, *instruction* and *musicality*, revealed – in comparison with the simpler model – that for both nPVIs (SOI and intensity) the general pattern of results for musicality was not affected by the inclusion of *instruction*, even though the two confounding variables were included in the model.

All musically active individuals in our subset were women. Therefore, our musicality variable could also be confounded by the factor gender.

One might criticize a missing control variable, since no data was collected for poem reading of non-manipulated poems, which then could have been used to compare reading for SOIs and intensity with ‘tack’ positions. However, we used the lines without tacks as baseline for comparison. Nevertheless, an updated version of the experiment should consider using originals, too.

## 5. Conclusion

At the syllable level, our results strongly suggest that both SOIs and intensities are used to mark stress differences with respect to a meter, but differently for musically active and musically inactive readers. With respect to nonsensical syllables such as tacks, musically active readers seem to maintain a prominence marking, but by using intensity. Musically inactive readers, on the other hand, experience a clash between top-down and bottom-up information. The nPVI results suggest a decrease in top-down processing for tacks, and even more so for musically active readers. However, the syllable-level results suggest that for tacks, musically active readers shift stress marking from SOI to intensities. The nPVI appears to capture metrical rhythmicity in the oral reading of conventional poetry. However, it fails to capture fine-grained processes at the syllable level.

In summary, our results suggest that the phonetic structure of syllables within the rhythmic ‘gestalt’ of a poem is indeed important for extracting, updating and maintaining a guiding metrical grid. We found that musically active participants tended to maintain the rhythmic structure better than inactive participants. Our findings contribute to the discussion of the iambic-trochaic law and the integration of bottom-up information in the predictive processing of language.

## Data availability statement

The raw data and the R script for data analysis supporting the conclusions of this article will be made available by the authors upon email request.

## Ethics statement

Ethical review and approval was not required for the study on human participants in accordance with the local legislation and institutional requirements. The patients/participants provided their written informed consent to participate in this study.

## Author contributions

JB and LK designed the experiments and contributed to data analysis and writing process. JB created the stimuli, conducted and ran the experiments. LK led the process. All authors made a substantial, direct and intellectual contribution to the article and approved the submitted version for publication.

## Funding

We acknowledge support by the Open Access Publication Fund of the University of Freiburg as well as support from the Institute of Psychology, Center for Cognitive Science, University of Freiburg, Germany.

## Conflict of interest

The authors declare that the research was conducted in the absence of any commercial or financial relationships that could be construed as a potential conflict of interest.

## Publisher’s note

All claims expressed in this article are solely those of the authors and do not necessarily represent those of their affiliated organizations, or those of the publisher, the editors and the reviewers. Any product that may be evaluated in this article, or claim that may be made by its manufacturer, is not guaranteed or endorsed by the publisher.

## Footnotes

^1^Written by the first author.

^2^ https://cspeech.ucd.ie/Fred/nPVI.php
